# Strategies for Tailoring Patient-Centered Technologies Across the Cancer Continuum: Protocol for a Scoping Review

**DOI:** 10.2196/73705

**Published:** 2025-09-09

**Authors:** Willi L Tarver, Diamond A Boyd, Pallavi Jonnalagadda, Mireille M Bitangacha, Timothy M Pawlik, Elizabeth Palmer Kelly, Electra D Paskett

**Affiliations:** 1 Division of Cancer Prevention and Control, Department of Internal Medicine College of Medicine The Ohio State University Wexner Medical Center Columbus, OH United States; 2 The Ohio State University Comprehensive Cancer Center The Ohio State University Columbus, OH United States; 3 The Center for the Advancement of Team Science, Analytics, and Systems Thinking in Health Services and Implementation Science Research (CATALYST) College of Medicine The Ohio State University Columbus, OH United States; 4 Department of Agricultural and Life Sciences College of Science Technology Engineering and Agriculture Central State University Wilberforce, OH United States; 5 Department of Surgery The Ohio State University Wexner Medical Center Columbus, OH United States

**Keywords:** scoping review, health information technology, tailoring, cancer continuum, underserved populations

## Abstract

**Background:**

In the United States, cancer is more prevalent in racial and ethnic minority groups and in rural-dwelling and low-income people. Compared with White people of non-Hispanic descent, Black and African American people have higher cancer mortality and Hispanic people are more likely to be diagnosed with infection-related cancers. In addition, people who live in persistent poverty areas are more vulnerable to cancer mortality. Tailoring health information technologies (HITs) can help bridge health inequities by providing these populations with relevant health information and cancer care. Cultural tailoring in health care involves adapting interventions to reflect a population’s values, history, and attitudes that influence behavior.

**Objective:**

The goals of the current study are as follows: 1) to understand what elements of tailoring HITs are most effective among different underserved populations, 2) to identify ways of incorporating these elements to improve the acceptability and effectiveness of technology-based interventions, and 3) to develop a framework to tailor HITs to underserved populations and improve engagement and acceptability.

**Methods:**

A scoping review will explore how HITs have been culturally tailored to underserved populations using PubMed, Scopus, and Web of Science database searches. Our search strategy will include terms and medical subject headings associated with the categories of cancer, HITs, tailoring, and underserved populations. We will also perform a snowball search of the references of included studies. We will include quantitative and qualitative peer-reviewed, English-language studies from the United States that examine efforts to tailor HIT interventions to improve their acceptance, use, and usability among underserved populations. Predefined inclusion and exclusion criteria will be applied for study selection. For each included study, we will extract the following data: study design, cancer type, underserved population of interest, details of the technology used, study methods, sample size, study outcomes, user acceptability, and tailoring and targeting strategies. The data will be summarized descriptively and analyzed thematically.

**Results:**

Preliminary searches following this strategy yielded a total of 784 citations (after removing duplicates) that will each be reviewed by at least 2 reviewers for inclusion. This protocol was submitted before data collection. The search strategy, citation screening, and data extraction will commence in accordance with the PRISMA-ScR (Preferred Reporting Items for Systematic Reviews and Meta-Analyses extension for Scoping Reviews) guidelines and the published protocol. Findings will be expected by the spring of 2026.

**Conclusions:**

There is a need to develop more accessible HITs for underserved populations. This scoping review will inform researchers, providers, and developers working on cancer-specific HITs for underserved populations, such as racial and ethnic minority groups, rural-dwelling residents, and low-income populations. By summarizing evidence on tailoring strategies by population and delivery mode, the review aims to support the development of more effective and acceptable technologies.

**International Registered Report Identifier (IRRID):**

PRR1-10.2196/73705

## Introduction

### Background

In the United States, over 18 million Americans are cancer survivors [1]. Although cancer is a disease that can affect anyone, some populations are more at risk of developing and dying from cancer than others. In particular, individuals who identify as being part of an underrepresented racial or ethnic minority group, live in a rural geographic area, or belong to a lower socioeconomic status have the highest risk of cancer. For example, African American men and women have higher cancer mortality and a lower 5-year survival after diagnosis compared with White men and women of non-Hispanic descent [2]. Additionally, compared with White individuals of non-Hispanic descent, Hispanic or Latino people are more likely to be diagnosed with cancers caused by infections [3]. Together, these findings underscore the urgent need for targeted interventions to reduce cancer disparities and improve cancer-related outcomes within these populations.

Various forms of health information technology (HIT) may offer a way to reach underserved populations. HIT is often defined as the “application of information processing involving both computer hardware and software that deals with the storage, retrieval, sharing, and use of health care information, data, and knowledge for communication and decision making” [4]. As such, it commonly refers to provider-facing electronic systems such as electronic health records, patient-facing personal health records, and other tools such as computerized provider order entry. However, there are other patient-facing technologies (hereafter referred to as patient-centered technologies) such as telehealth, mobile health (mHealth) apps, and patient portals [5]. These patient-centered technologies provide novel and promising ways to educate healthy individuals and patients with cancer on topics ranging from cancer prevention to managing a cancer diagnosis. These technologies are not always developed, however, with the populations of interest in mind. As a result, there is mixed evidence on how effective these technologies are among these populations [5]. Several factors influence the acceptability of HITs among patients with cancer, including age, educational achievement, and access to the internet [6]. For example, while mobile devices can help patients coordinate their health care through text message reminders, a systematic review conducted by Tarver et al [5] noted that underserved populations such as Black patients with cancer are not always up to date on how to use text messaging. Factors such as this can impact the effectiveness of these technologies and highlight the importance of bettering our understanding of different populations and the factors to consider when developing and implementing interventions designed to reach them.

An important and understudied element of intervention design is tailoring technologies based on elements such as the race, ethnicity, and language with which people identify. Tailoring is defined as any combination of techniques and information designed to target a single person based on traits that are unique to that person and connected to the desired outcome that is generated through an individual evaluation [7]. As an example, compared with a lay health advisor intervention, a tailored print and video intervention increased multiple colorectal cancer prevention efforts, such as fruit and vegetable consumption, recreational physical activity, and fecal occult blood testing screening, among a sample of African American church members [8]. This highlights the potential of tailored interventions in the prevention and early detection of cancer.

While the existing literature has largely focused on tailoring printed materials, less attention has been paid to the tailoring of HITs. A study done by Vilaro et al [9] noted that virtual health assistants tailored to Black women increased cancer screening in that community. While race and ethnicity are important elements to examine, culture is another essential component. Racial and ethnic minority populations may be distrustful of formal health services, contributing to their reliance on previously acquired cultural beliefs about health care [10-12]. An awareness of these barriers can aid in the development of interventions to help mitigate challenges and better engage these patient populations.

Furthermore, most studies demonstrated that the incorporation of HIT was linked to increased cancer knowledge; however, common shortcomings included a lack of language adjustment and tailoring to specific populations. For example, one study reported that a specific multimedia program (CancerHelp) [13] improved patient care for underserved populations by increasing their cancer knowledge. However, the program did not have a Spanish language version, thus excluding Spanish-speaking individuals who lack English proficiency. This issue highlights the need for researchers and care providers to work with developers to design interventions with the populations of interest in mind. When stakeholders take the needs and perspectives of these populations into consideration during the implementation of HITs, they are able to design solutions that lead to increased engagement [14]. Understanding how a population’s culture influences health behavior is necessary to develop HITs.

The purpose of the current study will be to systemically review current evidence on the tailoring of patient-centered technologies to underserved populations in the context of cancer care.

### Research Question

This review will seek to answer the question, “How can patient-centered technologies be tailored to engage underserved populations across the cancer continuum?” More specifically, we are interested in understanding the following aspects:

1. The importance of tailoring among underserved populations.
2. What elements of tailoring are most effective for different populations and at different points along the cancer continuum.
3. How tailoring can be used to address issues and barriers faced by underserved populations.
4. How to identify strategies to improve engagement.

## Methods

### Overview

This scoping review protocol was informed by the methodological framework developed by Arksey and O’Malley [15]. This framework guided the following steps: (1) identifying the research question, (2) identifying relevant studies, (3) study selection, (4) data charting, and (5) collating and reporting results [15]. The scoping review methodology was chosen as it is ideal for mapping a body of literature on a topic and identifying knowledge gaps [16]. We present steps 1 through 5 of our scoping review below.

### Step 1: Identifying the Research Question

As mentioned previously, the main research question that will guide our scoping review is, “How can patient-centered technologies be tailored to engage underserved populations across the cancer continuum?” Our scoping review will aim to extract information related to tailoring technologies for different populations along the cancer continuum and synthesize the available evidence, thereby creating a framework that can be used to develop or enhance technologies seeking to better engage populations of interest.

### Step 2: Identifying Relevant Studies

#### Search Strategy

The database search will be conducted using the electronic databases PubMed, Scopus, and Web of Science. These databases were chosen because they index and abstract the major health and medical journals and scholarly literature. Keywords were developed from existing literature in addition to medical subject heading (MeSH) terms to identify eligible studies. Keywords will include relevant terms corresponding to categories related to the disease class of cancer, underserved populations, cultural tailoring or targeting, and types of HITs. A full list of the keywords can be found in Table 1. In addition, we will perform a snowball search method by reviewing the references of the final list of included studies to ensure all relevant studies were captured.

**Table 1:** Operationalization of the search terms. Search terms within each category will be combined with OR. Search terms between categories will be combined with AND.

Category	Search terms
Cancer	Cancer*^a^ OR neoplas* OR carcino* OR onco* OR malignan* OR tumour OR tumor OR hematolog* OR haematolog* OR lymphoma OR leukemia OR leukemia OR leukaemia
Underserved populations	“Minority Health” OR “Healthcare Disparities” OR “Health Status Disparities” OR Poverty OR “low income” OR Ethnic* OR race OR racial OR disparit* OR minorit* OR underserved OR underrepresented OR inequalit* OR rural OR hispanic* OR mexican* OR latin* OR african* OR black* OR asian OR indian* OR alaskan* OR “alaska* native” OR “native american*” OR inuit OR “pacific islander” OR immigrant
Tailoring	personaliz* OR personalis* OR individualiz* OR individualis* OR tailor* OR customiz* OR customis* OR “linguistically target*” OR “culturally target*” OR “patient target*”
Patient-centered health information technologies	“health information technology” OR “health it” OR “electronic health records” OR “electronic health record” OR “electronic medical record” OR “personal health record” OR “personal medical record” OR “patient accessible record” OR “patient portal” OR “patient internet portal” OR “decision support” OR “clinical reminder” OR “electronic reminder” OR “reminder system” OR “m-health” OR “mhealth” OR “mobile technolog*” OR “mobile health” OR “cell phone” OR “cellular phone” OR “smartphone” OR “mobile phone” OR “mobile device” OR “text message” OR “cd-rom” OR “dvd” OR “computer based” OR “computer-based” OR “internet-based” OR “web-based” OR “web based” OR “e-health” OR “ehealth” OR “tablet” OR “telemedicine” OR “telehealth” OR “teleoncology” internet

^a^The asterisk (*) denotes truncated terms. For example, minority groups identified as being underserved will be captured using the following truncated keywords: black* and african* for Black or African American groups and hispanic*, mexican*, and latin* for Hispanic or Latino groups.

### Definitions

#### Patient-Centered Technologies

Patient-centered technologies are HIT applications that are patient-facing, making them “well suited to engage patients, promoting a more active role for patients in health care” [17]. For the purposes of this review, we are interested in including studies using personal health records or patient portals, mHealth, telehealth, and the Internet and access to websites.

#### Tailoring

Cultural tailoring is defined as any combination of information intended to reach one specific person based on characteristics unique to that person related to an outcome of interest derived from a personal assessment [7].

#### Targeting

Targeting is defined as the development of interventions for a defined population subgroup. This approach takes into account the various shared characteristics, such as culture, of the subgroup’s members [18].

#### Underserved Population

Medically underserved populations are groups who may face economic, cultural, or linguistic barriers to medical care services [19]. For the purposes of this review, we will focus on racial and ethnic minority groups, individuals of lower socioeconomic status, and people residing in rural geographic areas.

#### Inclusion and Exclusion Criteria

We will include peer-reviewed journal articles published in the English language and studies conducted in the United States. We will include both qualitative and quantitative studies for insights on patient perspectives as well as the effectiveness of the interventions. In addition, we are interested in studies that explore efforts to tailor or target the design of interventions to improve their acceptance, use, and usability among underserved populations.

Conversely, we will exclude nonempirical studies such as commentaries, editorials, letters-to-the-editor, conference abstracts, and protocols. In addition, we will exclude international studies due to sociocultural differences across countries. We will also exclude other reviews. If the search identifies other reviews on similar topics, we will, however, search their reference lists as part of our snowball strategy to identify additional articles.

### Step 3: Study Selection

Studies will be selected using a 2-step inclusion process. The title and abstract for each citation will be reviewed in step 1. If the title or abstract is classified as a systemic review or protocol, does not incorporate HITs, does not focus on cancer, is conducted outside of the United States, or is published in a language other than English, the study will be excluded. In step 2, the full-text articles of the remaining citations will be retrieved for review. Similarly, we will exclude citations that do not meet our inclusion criteria.

During the review process, 2 reviewers will review each citation’s title and abstract (step 1) and full-text article (step 2). The use of 2 reviewers has been found to increase the reliability of article selection. When the inclusion criteria are unclear during title and abstract review, reviewers will err on the side of inclusion during step 1, progressing it to step 2. A full-text review will then determine eligibility. For example, if an abstract met the criteria of using HIT to improve breast cancer knowledge among American women, but did not specify the inclusion of underserved individuals, it would not be excluded. It would move forward to the next step so that the full-text article could be reviewed to ascertain eligibility.

### Step 4: Data Charting

A team of 4 reviewers (WT, DB, PJ, and MB) will perform the data charting process with each article being reviewed and extracted in pairs. A standardized form will be developed and used to extract data from the included studies for evidence synthesis. Systematically extracted information will include the following information from each included study: study design, cancer type, the underserved population of interest, the type of technology used, details of the technology used, study methods, sample size, study outcomes, indicators of user acceptability, and methods used to tailor and target interventions. Additional variables may be added during the data collection and analysis phases. Two reviewers will independently extract data from each study. Disagreements will be resolved through discussion with the study team. If needed, missing information relevant to our review will be requested from the studies’ authors.

### Step 5: Collating and Reporting Results

After extracting data as described in step 4, we will create descriptive and numerical summaries of the studies that meet our inclusion criteria. Study characteristics will be presented in a tabular form and narratively summarized within the text. Gaps in the literature will be identified by using comparative analysis of study participant characteristics, study designs used, and HITs studied.

The qualitative data that we extract pertaining to tailoring effects will be subjected to thematic analysis according to the inductive-deductive approach described by Clarke and Braun [20]. This approach will guide us to identify themes and commonalities in how HIT is tailored. As a team, we will discuss and develop these descriptive themes until we reach consensus. As we present our findings, we will also highlight similarities and differences in how HIT is tailored for different populations of interest. In summary, we will follow a three-stage process including the following steps: (1) coding of the findings and results of the included studies, (2) organization of the codes into related areas, and (3) development of analytical themes. These data will be descriptively summarized in tables by theme and narratively reported.

This scoping review will synthesize both quantitative and qualitative research. As a result, a meta-analysis will not be feasible due to the expected variability in study designs, methodologies, and outcome measures.

## Results

This protocol was submitted before data collection. The search strategy, citation screening, and data extraction will commence in accordance with the PRISMA-ScR (Preferred Reporting Items for Systematic Reviews and Meta-Analyses Extension for Scoping Reviews) guidelines [21] and the published protocol. Findings will be expected by the spring of 2026. A preliminary search produced a total of 1395 articles. Duplicate records (n=221) were removed, leaving a total of 784 unique articles. The inclusion and exclusion criteria will be strictly followed in performing the forthcoming study selection. The data will be summarized narratively to assess the elements of tailoring patient-centered technologies to underserved populations.

## Discussion

### Rationale for the Study

To our knowledge, this is the first scoping review examining the tailoring of patient-centered technologies for underserved populations across the cancer continuum. Despite the widespread use of patient-centered technologies for health, research in this area is limited. This scoping review aims to address this important knowledge gap by synthesizing existing evidence and identifying areas for future research. A clear understanding of which tailoring methods can be used in the development of patient-centered technologies will enable effective decision making by policymakers, researchers, clinicians, and developers. In addition, it will provide these stakeholders with a framework for understanding the most effective methods and modes of delivery to use for specific populations of interest. Thus, this review will inform the creation of more effective and engaging tools. These results will further extend the knowledge gained from a previous systematic review, which found that patient-centered technologies seem to be more effective when tailored to the populations of interest [5]. This protocol was informed by established PRISMA-ScR guidelines to conduct systematic and scoping reviews [21,22].

### Strengths and Limitations

Given the breadth of this proposed scoping review, we anticipate challenges and have outlined mitigation strategies in this protocol. The first challenge relates to the inconsistent language used in studies that may incorporate tailoring in their development process. We constructed our search strategy to address this by expanding it to include keywords related to targeted interventions and personalizing or customizing interventions.

Second, we anticipate limited descriptions of patient-centered technologies and how they were developed and tailored. Authors may not adequately detail the development and tailoring process of their technologies. If needed, we will contact study authors with the intent of obtaining additional information to mitigate this challenge.

### Conclusions

When implementing patient-centered technologies for underserved populations, cultural backgrounds should be considered along with effective methods of tailoring and modes of delivery. This review will aim to present a summary and analysis of the existing evidence on tailoring HITs to underserved populations. We believe this review will be an invaluable aid and reference to researchers, providers, and developers aiming to develop or enhance cancer-specific HITs for racial and ethnic minority groups, populations residing in rural areas, and people with low income. The result of this scoping review of cancer-specific patient-centered technologies will be a detailed summary of the evidence for tailoring these technologies organized by underserved populations of interest and modes of delivery. By providing a structured summary of the evidence base for cancer-specific patient-centered technologies, this review aims to inform and facilitate studies developing and implementing these technologies, ultimately contributing to their improved acceptance and effectiveness.

## Appendix

Multimedia Appendix 1 PRISMA-ScR checklist.

## Abbreviations

HIT: health information technology
MeSH: medical subject heading
mHealth: mobile health
PRISMA-ScR: Preferred Reporting Items for Systematic Reviews and Meta-Analyses Extension for Scoping Reviews
